# Evaluating the Histologic Grade of Digital Squamous Cell Carcinomas in Dogs and Copy Number Variation of KIT Ligand—A Correlation Study

**DOI:** 10.3390/vetsci10020088

**Published:** 2023-01-24

**Authors:** Argiñe Cerezo-Echevarria, Alexandra Kehl, Christoph Beitzinger, Tobias Müller, Robert Klopfleisch, Heike Aupperle-Lellbach

**Affiliations:** 1 Pathology Department, LABOKLIN GmbH & Co. KG, 97688 Bad Kissingen, Germany; 2 Institut für Bioinformatik, Universität Würzburg, 97070 Würzburg, Germany; 3 Institute of Veterinary Pathology, Freie Universität Berlin, 14163 Berlin, Germany

**Keywords:** canine, cancer, toe, grading, haircoat, color, genetics, gene

## Abstract

**Simple Summary:**

Dark-haired dogs are predisposed to the development of digital squamous cell carcinoma (DSCC), suggesting an underlying genetic predisposition which is yet to be explained. Some authors have suggested a correlation between the number of copies of KIT Ligand, a gene associated with cell survival, proliferation, and melanogenesis, among other functions, and the potential predisposition to DSCC in dogs. This was evidenced by the fact that dogs with DSCC had a significantly higher copy number of this gene than those who did not have the neoplasia. For this reason, we evaluated the potential correlation between the number of copies of the KIT Ligand in genomic DNA with the histological grade of malignancy in dogs with DSCC. Our findings reveal a significant correlation between the number of copies of KIT Ligand and DSCC histological grade. This supports previous studies that KIT Ligand may play a role in DSCC development and, additionally, may be involved with the presence of histologically malignant morphological features. This suggests a potential factor in the development of canine DSCC, which may signify a potential advance in personalized veterinary oncological approaches as well as future breeding programs.

**Abstract:**

Dark-haired dogs are predisposed to the development of digital squamous cell carcinoma (DSCC). This may potentially suggest an underlying genetic predisposition not yet completely elucidated. Some authors have suggested a potential correlation between the number of copies KIT Ligand (KITLG) and the predisposition of dogs to DSCC, containing a higher number of copies in those affected by the neoplasm. In this study, the aim was to evaluate a potential correlation between the number of copies of the KITLG and the histological grade of malignancy in dogs with DSCC. For this, 72 paraffin-embedded DSCCs with paired whole blood samples of 70 different dogs were included and grouped according to their haircoat color as follow: Group 0/unknown haircoat color (*n* = 11); Group 1.a/black non-Schnauzers (*n =* 15); group 1.b/black Schnauzers (*n =* 33); group 1.c/black and tan dogs (*n =* 7); group 2/tan animals (*n =* 4). The DSCCs were histologically graded. Additionally, KITLG Copy Number Variation (CNV) was determined by ddPCR. A significant correlation was observed between KITLG copy number and the histological grade and score value. This finding may suggest a possible factor for the development of canine DSCC, thus potentially having an impact on personalized veterinary oncological strategies and breeding programs.

## 1. Introduction

The largest reported cohort of 2912 canine toes, conducted by Grassinger et al., classified 52% of their samples as neoplasms, 78% of which were malignant. Of those, approximately 65% were digital squamous cell carcinomas (DSCCs) [1]. These findings, along with other similar research [2,3,4,5], strongly suggest that DSCC represents the most common malignant tumor at this location in the canine population [1,5,6].

This aforementioned study, coinciding with other publications [6,7,8,9,10], identified a DSCC predisposition in dark canine breeds such as Schnauzers [7,8,10], Briards [7], Rottweilers [6], Poodles [7,9], and Dachshunds [6]. On the other hand, interestingly, Grassinger et al. [1] observed that Jack Russell Terriers, which commonly have white paws [11], were less likely to develop the neoplasia at this location than other mixed breeds [1]. These peculiar differences, apparently based on haircoat color, along with publications of three related Schnauzers with primary DSCC [10], may suggest an underlying genetic predisposition for the development of this neoplasia based on the dark haircoat.

KIT Ligand (KITLG), also known as stem cell factor [12], is a gene that encodes the ligand of the receptor-type protein-tyrosinase kinase KIT. In general terms, this is a low specific ligand that is associated with many essential vital roles, such as cell survival and proliferation, hematopoiesis, stem cell maintenance, gametogenesis, mast cell development, migration, and function, and melanogenesis [12,13,14]. It is for this last feature that KITLG is a significant gene in canine haircoat color and tone determination. This is because it plays a role in postnatal cutaneous melanogenesis and follicular epithelial melanocyte terminal differentiation [15]. The melanin synthesis of the hair follicle’s melanocyte is achieved through an intercellular signaling pathway. This results in the production of both eumelanin and pheomelanin by the melanosome, a specialized organelle, along the entire extension of the hair shaft, thus conferring different colors and tones [15,16]. Interestingly, Bannasch et al. [15] stated that the copy number of the CNV of KITLG was significantly associated with eumelanin intensity in the Poodle and across breeds and, to a lesser extent, pheomelanin.

Moreover, Karyadi et al. [7] suggested that a higher CN of the KIT ligand locus is seen in dogs with DSCC when compared to animals without the tumor. This new finding, along with the known role of KITLG in haircoat melanogenesis and the findings of Bannasch et al. [15], may suggest a potential link between the presence of a high CN of the KITLG and the development of a DSCC in dark-haired dogs. This generated the hypothesis that a higher KITLG count may not only be associated with the development of DSCC, but potentially also with a morphologically distinct DSCC which could correlate with a more aggressive histological grade. To the best of the authors´ knowledge, there are no studies correlating the CN of KITLG with distinct histological characteristics of canine DSCC.

The objective of this research was to evaluate if there is a correlation between histological features of aggressiveness in canine digital SCC and copy number values in KITLG. 

## 2. Materials and Methods

From 2014–2019, 72 histological samples of DSCC from 70 dogs were selected retrospectively from the routine diagnostic pool submitted to LABOKLIN GmbH & Co. As a selection requirement, the samples had to contain a clear neoplastic invasive front and known breed. All DSCC from animals only included fragments with neoplasia, and samples with no clear invasive front or without information about the breed were excluded. 

Furthermore, additional blood samples from these dogs were available from routine diagnostics (presurgical or geriatric screening). As all samples (toes and whole blood) were submitted for routine diagnostic purposes, ethics committee approval was not required (RUF-55.2.2-2532-1-86-5). All the material used was no longer needed for diagnostics.

The ages of the dogs ranged from 4 to 16 years, with a median of 10 years. Sex was either female intact (15), female spayed (15), male (22), or male castrated (20). Limb and toe affected, when available, was noted. The breeds included Giant Schnauzer (20), Standard Schnauzer (14), Mixed (8), Briard (5), Labrador (5), Gordon Setter (3), Russian Black Terrier (2), Flat-Coated Retriever (2), Bernese Mountain Dog (2), Rottweiler (2), Belgian Shepherd (1), Havanese (1), Irish Setter (1), Puli (1), German Wirehaired Pointer (1), Hovawart (1), Giant Poodle (1), West Highland White Terrier (1), and Standard Poodle (1).

The majority of these animals were further grouped according to their haircoat color, similar to the previous research [17], as follows; Group 0/others (*n =* 11), composed of animals with unclear haircoat color; Group 1.a/blacks (*n =* 15), with non-Schnauzer black breeds; Group1.b/Schnauzers (*n =* 33), with black Schnauzers; Group 1.c/black and tan (*n =* 7), with black and tan animals; and Group 2/light (*n =* 4), with dogs with a light blond or reddish haircoat. Additionally, Groups 1.a, 1.b, and 1.c were summarized as “dark coated breeds” (*n =* 55). Signalment and individual cases with more detailed information can be seen in Supplementary Table S1.

Similar to the previous study conducted by this research group [17], all digital samples were fixed in 10% phosphate-buffered formalin, routinely trimmed following laboratory standard procedures, and decalcified in a mixture of ≥10– <20% hydrochloric acid (HCl) and formaldehyde (≥3%–<5%) (Osteomoll^®^ rapid decalcifier solution for histology; catalogue no. 101736) over a period of 24–72 h, periodically assessing tissue until it was ready to be further processed according to published trimming guidelines [18]. Afterwards, longitudinal and sagittal sections were embedded in paraffin wax and cut at 4–5 µm thickness to be then stained with Haematoxylin–Eosin (HE). All slides were reviewed, selecting the most representative section for this study. This was based on a good histological quality and clear invasive front with surrounding non-affected stroma to evaluate the neoplastic–non-neoplastic transition. The most representative slide was scanned and analyzed through specialized image analysis software (NIS-elements software (Nikon, Tokyo, Japan); Aperio ImageScope (Leica, Wetzlar, Germany)) by a blinded ACVP diplomat (A.C-E).

### 2.1. Histological Examination

For more detailed information concerning the adapted gradings performed in the study, please refer to the previous open-source publication carried out by this research group (Cerezo-Echevarria et al. 2020) [17].

#### 2.1.1. Invasive Front Grading System (IFGS)

**Figure 1:**
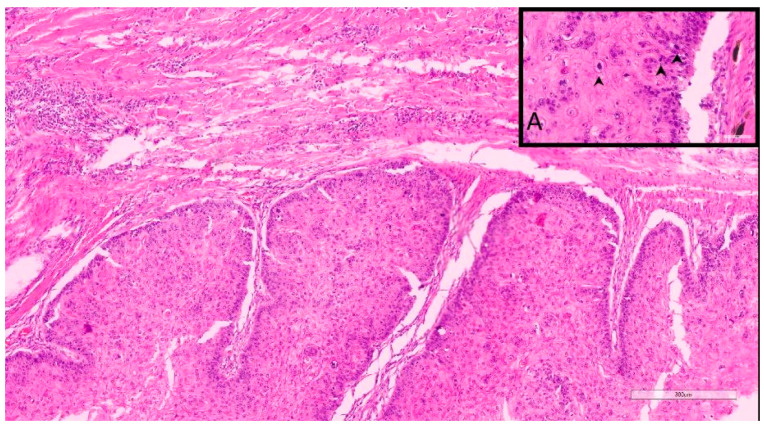
Sample 11, 9-year-old, female spayed, Caucasian Shepherd mix. This DSCC was graded as I by both Invasive Front and Tumor Cell Budding Systems. **Inset A**. Note that despite the low grade, there are occasionally moderate numbers of mitotic figures (arrows).

**Figure 2:**
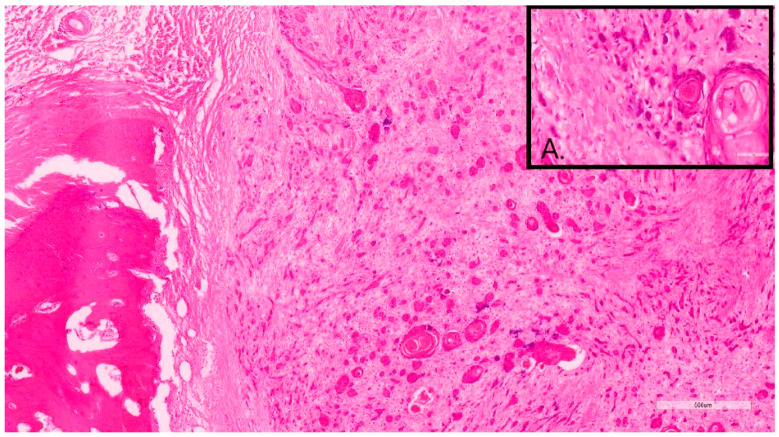
Sample 12, 11-year-old, female, Briard. This DSCC was graded as grade III by both Invasive Front and Tumor Cell Budding systems. **Inset A.** Note the marked neoplastic cellular dissociation within the invasive front, often forming small neoplastic buds or individual cells.

As previously performed [17], Nagamine et al.’s (2017) [19] grading system for canine oral SCC (OSCC) was adapted, following the established criteria of degree of keratinization, pattern of invasion, host response, nuclear pleomorphism, and mitoses per high-power field (HPF) (Table 1). The mitoses per 10 HPF were assessed in an overall area of 2.37 mm2 to ensure standardization [20] and reproducibility. The final addition of the score values of these five morphologic features resulted in a total IFGS score value, which then was translated into 3 different grades, including well-differentiated/grade I (total score value: 6–10) (Figure 1), moderately differentiated/grade II (total score value: 11–5), and poorly differentiated/grade III (total score value: 16–20) (Figure 2) [17,19].

#### 2.1.2. Tumor Cell Budding Grading System (TCBGS)

In a similar manner to previously conducted research by this group [17], an adaptation from two similar human SCC grading systems of Jesinghaus et al. (2018) [21] and Boxberg et al. (2019) [22] was used. In this grading, individual features of invasive front tumor budding in 10 HPF, smallest nest size, and stromal response associated with the neoplasm were evaluated. 

Tumor budding was considered when neoplastic aggregates/complexes fewer than 5 cells disconnected from the main neoplasm and infiltrated into the surrounding stroma. These “buds” were assessed in an area of 2.37 mm2 at 40x magnification in high-incidence areas. The point system was granted following the table (Table 2). The sum of the score of each evaluated feature results in a final score value that translated into 3 different grades, including well-differentiated/grade 1 (total score value: 2–3) (Figure 1), moderately differentiated/grade 2 (total score value: 4–5), and poorly differentiated/grade 3 (total score value: 6–7) (Figure 2) [17,21,22].

### 2.2. Genetic Analysis

Genomic DNA extraction and isolation from EDTA blood were performed with the MagNA Pure 96 system using DNA Tissue Lysis Buffer and viral NA Small RNA kit (Roche, Basel, Switzerland) according to manufacturers´ manual. Similar to that described by Bannasch et al. [15], the copy number quantification of the KITLG CNV was performed with digital droplet PCR (ddPCR) using TaqMan^®^ assays specific for the KITLG CNV sequence and proto-Oncogene 1 (ETS1) as reference gene. Measurement was carried out in duplicate, and the mean value was used for further analyses. Intra-assay correlation was 0.85. Copy number was determined using DropletReader (Bio-Rad, Feldkirchen, Germany) and QuantaSoftware (Bio-Rad, Feldkirchen, Germany). 

### 2.3. Statistical Analysis

Statistical analysis between dark- (Group 1, n = 55) and light-coated (Group 2, n = 4) breeds as well as between the different pre-established subgroups (1.a/blacks, n = 15; 1.b/Schnauzers, n = 33; 1.c/black and tan, n = 7; 2/light, n = 4) were performed using the Mann–Whitney U test. Group 0 was excluded because of heterogenic genetic background.

All statistical analyses and visualizations were carried out with the statistical framework R version 4.2 (R Core Team 2022). To identify the optimal model, we applied the “step“ R function with direction “both“ and the Bayesian Information Criterion (BIC). The marginal effects of the interaction term were visualized by the function “plot_model” as implemented in the “sjPlot” R package Lüdecke [23]. Robust linear regression was performed by the function “rlm” as implemented in R package MASS [24]. The correlation significance levels were calculated according to the “rob.pval” function implemented in the “repmod” R package [25].

## 3. Results

Out of the 72 samples from 70 different animals, 46 DSCCs were located in the forelimb and 24 in the hindlimb. Two samples were not provided with an exact location.

### 3.1. Histological Assessment

#### Histological Grades according to Groups 

Following a similar model to that provided in previous studies conducted by this research group [17], the samples of DSCC were graded twice according to both IFGS and TCBGS. The total score values for IFGS ranged from 5 to 19, while the TCBGS total score values ranged from 2 to 7. For further detail on the histological grade distribution of the canine population in general and the pre-established groups according to the IFGS and TCBGS, please refer to the previous research [17]. Interestingly enough, when comparing both systems simultaneously, twenty-three animals were classified as grade I, fourteen as grade II, and seven as grade 3 by both systems. As described in the previous study, there were single animals (a Giant Schnauzer and two Labrador Retrievers) classified as grade I with the IFGS, while being a grade 3 by the TCBGS (Supplemental Table S1, samples 4, 22, and 31). On the other hand, there were no cases simultaneously classified as a grade III by the IFGS while being grade I on the TCBGS. Two animals were reported to each have two digital squamous cell carcinomas (Supplemental Table S1, samples 25–26 and 42–43, respectively). The 10-year-old, male, intact Russian Black Terrier had one an intermediate grade DSCC according to both systems (II/2) (sample 25), while the second tumor was classified as a grade III/3 by IFGS and TCBGS (sample 26). The 11-year-old, female, intact Giant Schnauzer had one of the DSCCs classified as grade II/2 (sample 43), while the other tumor was grade II/1 (sample 42). 

**Figure 3:**
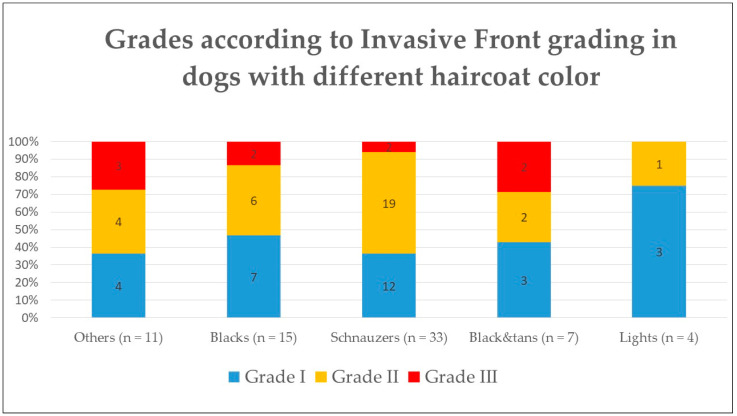
Histological grade according to Invasive Front Grading System in dogs according to their haircoat color.

When associating the IFGS grade with the haircoat color, there was no significant difference between any of the groups (Figure 3) or between color and the histological features evaluated such as mitoses, nuclear pleomorphism, keratinization, invasion, or host response (*p* > 0.5).

**Figure 4:**
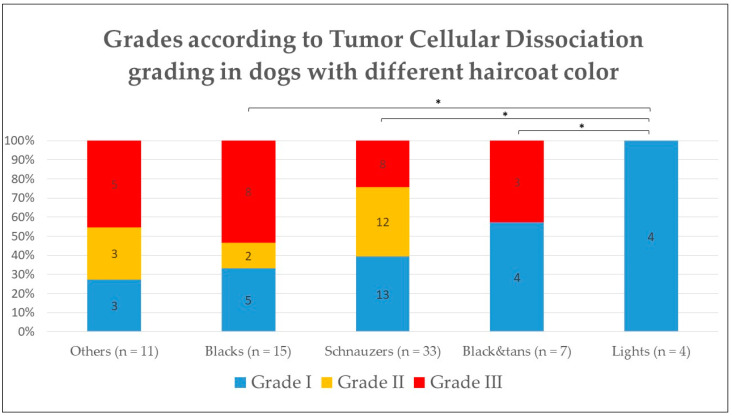
Histological grade according to Tumor Cellular Dissociation Grading System in dogs according to their haircoat color. * *p* < 0.05.

On the other hand, when comparing the TCBGS grade with the haircoat color, there were significant differences between group 2/light and the other groups (1.a, 1.b, 1.c) (*p* < 0.05), with group 2 having a lower grade that any of the other darker groups (Figure 4). However, there was no significant difference when evaluating each individual feature that composed the TCBGS grade, including smallest nest size, mitoses, and tumor budding activity. 

In summary, no particular histological feature is significantly different between the dark and light groups, but the overall summary of different histological aspects (as those evaluated on the TCBGS) may result in significant statistical differences in the degree of tumor differentiation and potential malignant behavior. 

### 3.2. Copy Number Variation on KITLG

The value of the copy number (CN) of KITLG was measured for each animal included in the study and ranged between 2.02 and 9.14, with a mean value of 5.6. The value of Group 0/others was 5.7, for Group 1.a/black it was 5.8, for Group 1.b/Schnauzers it was 5.7, for Group 1.c/black and tan it was 5.5, and for Group 2/light it was 4.5. The differences between the groups were not significant.

A statistical analysis comparing the independent correlation of the TCBGS grade and IFGS grade with the KITLG copy number (CN) value was performed. Comparatively, animals with IFGS grade I had significantly lower KITLG CN than those of grade II (*p* = 0.001) and grade III (*p* = 0.007). On the other hand, when comparing KITLG CN with TCBGS, only animals with grade III had a significantly higher KITLG CN value than those animals with grade I (*p* = 0.042). This suggests that a higher histological grade is correlated with a higher KITLG CN value when comparing low versus high grades in both histological systems applied. 

**Figure 5:**
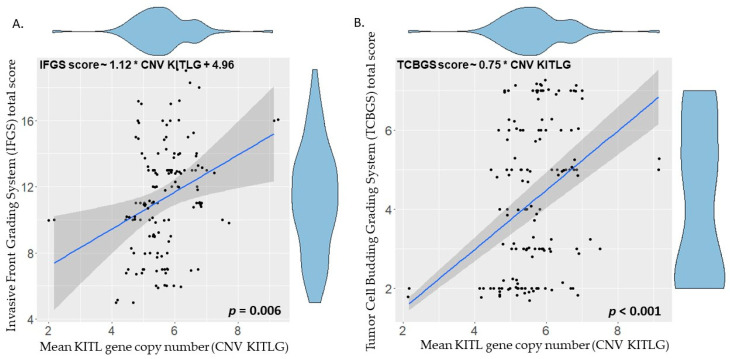
Linear regression of KITLG copy number (x-axis) and histological score value (y-axis) of the different animals. (**A**) Invasive Front Grading total score value (IFGS) is significantly correlated with the KITLG number (IFGS score ~ 1.12 * CNV KITLG + 4.96; *p* = 0.006). (**B**) Tumor Cell Budding Grading (TCBGS) total score value is significantly correlated with the KITLG number (TCBGS score ~ 0.75 * CNV KITLG; *p* < 0.001).

The optimal linear regression model was obtained using the R function “step” with direction “both” and the Bayesian information criterion (BIC). The identified optimal regression model considers only the KITLG CN value and the IFGS score value, with a strong significant correlation between these two features (IFGS score ~ 1.12 * CNV KITLG + 4.96; *p* = 0.006) (Figure 5A). The TCBGS score values and KITLG CN also show a significant correlation (TCBGS score ~ 0.75 * CNV KITLG; *p* < 0.001) (Figure 5B). These results suggest that there is a significant direct correlation between the evaluated histological score and the KITLG CN value. This model was independent from the haircoat color and/or gender. For further information regarding this correlation, taking into account each of the phenotypic haircoat colors, please refer to Supplemental Figure S1.

## 4. Discussion

In veterinary medicine, an effort has been made to try to identify genes associated with cancer or disease in different canine breeds or lineages. Some examples of this include renal cystadenocarcinoma [26], histiocytic sarcoma [27,28,29], progressive rod-cone degeneration [30], or collie eye anomaly [31], among others [32,33,34,35,36,37]. This can be particularly interesting, not only for tumor and disease occurrence predisposition, but also potentially its clinical behavior, prognosis, outcome, and future breeding decisions.

To this end, this represents the largest collection of canine DSCC along with pairing whole blood samples to date, allowing both histological and genetic assessment of the given animals. The aim of the study was to evaluate a hypothetical correlation between histological features of invasiveness in canine digital squamous cell carcinoma, based on two different adapted histological grading systems (IFGS and TCBGS), and copy number values of KITLG. Animals with lower IFGS and/or TCBGS score values had a significantly lower KITLG copy number (IFGS ~ 1.12 * CNV KITLG + 4.96; *p* = 0.006; TCBGS ~ 0.75 * CNV KITLG; *p* < 0.001).

As a continuation of the previous work conducted by this research group [17], the actual study confirms the hypothesis that KITLG CNV may have a potential role in the development of malignant histological features in DSCC. This is also backed up by the fact that in other studies, Schnauzers, which are often overrepresented, had worse outcomes against DSCC than other breeds [8]. This is despite not having identified poor prognostic factors at the time of presentation [8]. Furthermore, animals with multiple DSCCs have a tendency to develop additional ones over time [8]. This last remark, along with the description of DSCC in three related Giant Schnauzers [10], further implies the possibility of an underlying genetic predisposition, unknown at the time. On a different note, Bannasch et al. 2021 [15] found a correlation of CNVs in the KITLG color variation between pheomelanin and eumelanin deposition in the haircoat of the Poodle and other breeds. As expected, the present results are mainly based on a majority of eumelanin-based, dark-colored dogs of different breeds, and therefore, correlation between animalsdark and light haircoats and related CNV could not be established. Additionally, Grassinger et al. 2021 [1] already suggested that the absence of eumelanin (dark pigmentation) within the haircoat and/or claws may potentially have a protective function for light-haircoat dogs for the development of DSCC.

On a somewhat similar line of investigation, Karyadi et al. [7] suggested that a KITLG CNV of 4 or more may be the cause of a predisposition to the development of DSCC in the canine population, given that dogs with DSCC had a higher KITLG CN value than their non-DSCC counterparts. In the present study, the overall mean value of KITLG CN was 5.6, and all animals suffered from DSCC. Similar to that hypothesis, in the present study, all groups had a mean CN value of more than 4 (ranging from 4.5 in the light/2 group to 5.8 in the black/1.a group). There was only a single animal (sample 71, a 4-year-old, female, mixed-breed dog) which had a copy number of less than 4, being 2.17 in this case. This could support the hypothesis from Karyadi et al. that a KITLG CNV of 4 or greater predisposes the animal to DSCC. However, another option is that that this represents a widespread alteration in the CNV across several breeds. The dog with a CN of less than 4 in our study could possibly represent a spontaneous tumor development, with no genetic predisposition. 

Similar to previous studies [17], the degree of differentiation of DSCC in both dark- and light-haircoat canine populations was assessed, obtaining aligning results. When applying the TCBGS, there were more histological “malignant” features in darker breeds (Group 1) than in the lighter ones (Group 2) (*p* < 0.05). On the other hand, when looking into the IFGS, there were no significant differences between the grade and haircoat color group. Nonetheless, statistical interpretation should be cautious, given the large group size disparities. 

The KITLG copy numbers were compared independently to the histological score values of both the TCBGS and the IFGS systems of the established animal groups. Interestingly enough, both IFGS and TCBGS showed strong correlation between the KITLG CN and their histological score values. In summary, animals with a higher KITLG CN value have more histological features of malignancy of DSCC. IFGS had a slightly inferior correlation with the KITLG CN (*p* = 0.006) compared to TCBGS (*p* < 0.001), but both correlations were robust. The slight correlation discrepancy between grading systems and KITLG CN could be partially explained by the fact that both grading systems pay attention to different histological features, and potentially one of those features may be correlated with a higher (or lower) KITLG CNV. This has been established independent of the haircoat color and/or gender. It must be noted that this estimation may have been a more robust linear regression model if extreme mean CNV values would have been available. Despite this, with the available data, there was still a robust linear regression with comparable results. This may potentially suggest that with a known KITLG CN value, one could potentially predict the histological malignancy of a present DSCC, thus deciding the most appropriate course of treatment.

This study included 36 Schnauzers, known to be predisposed to DSCC, both Giant (n = 20) and Standard (n = 14). However, the present study did not compare animals of the same breed with and without DSCC, so a relative KITLG CNV could not be established between affected and non-affected animals. It is for this reason that we cannot draw conclusions regarding the comparatively higher KITLG CNV of animals with and without this tumor from the same breed. In order to prove this, further larger studies of dogs of the same breed (to ensure a more homogeneous sample) with and without DSCC in their entire lifetime should be performed to evaluate whether the hypothesis that a higher KITLG CNV is related to animals developing DSCC still stands. This is currently under investigation to allow future comparisons. Additionally, as a correlation between KITLG CNV and malignant histological features are observed, future studies should include KITLG transcription and protein expression level. Currently, there are few studies investigating the molecular expression of KITLG and c-KIT in veterinary medicine, mostly in canine mast cell tumors [38,39,40,41,42] and gastrointestinal stromal tumors [42,43,44], with no descriptions of specific immunolabeling in canine SCC or DSCC. In human medicine, there have been attempts to perform immunohistochemistry for c-KIT on SCC [45,46,47,48,49,50,51]. In the literature, there are reports that between 12.5% and 20% [45,46,47,50] of SCCs have any kind of sparse immunopositivity. In a single study, the expression of c-KIT in esophageal SCC was correlated with a worse prognosis, only being expressed in 29.9% of the specimens [48]. In a similar manner, c-KIT expression was associated with lymph node metastasis, histological type, and worse overall survival in human non-small cell lung cancer, according to a meta-analysis [49]. Interestingly enough, in this study, the expression of c-KIT in adenocarcinoma was higher than in SCC [49], perhaps associated with innate tumor malignancy and aggressiveness. This, however, was beyond the scope of the present study and future projects may include these further tests.

## 5. Conclusions

This study represents the largest of its kind pairing up canine DSCC with blood samples, allowing a simultaneous histological and genetic assessment. When assessing the results, there was a significant correlation between KILTG CN increase and higher histological score value on both IFGS and TCBGS.

### Further Studies

It would be interesting to evaluate a large cohort of canine breeds non-predisposed to DSCC to establish a KILG CNV baseline, identifying if there is a significant difference when these same breeds develop a DSCC. With this, KITLG CNV can be seen in a large cohort of animals with and without DSCC. More homogeneous groups, along with the identification of other “protective” or “causative” genes for CDSCC, should be evaluated. Further studies are needed to further characterize a possible genetic test for the predisposition of DSCC in dark-haired breeds, as well as KITLG transcription levels and final protein expression. This could possibly aid in identifying other potential genes that may serve as a protection against this neoplasm or may confer it with more benign or malignant histological features.

## Figures and Tables

**Table 1:** Simplified table of Invasive Front Grading System (IFGS) used in the present study of canine digital squamous cell carcinoma (adapted from Nagamine et al. (2017) for use in canine oral squamous cell carcinoma).

Morphological Feature	Score Value			
	1	2	3	4
Degree of keratinization	>50% cells keratinized	20–50% cells keratinized	5–20% cells keratinized	0–5% cells keratinized
Pattern of invasion	Pushing, well-differentiated borders	Infiltrating, Solid cords, bands and/or strands	Small groups of cells (*n* > 15)	Small groups and/or single cells (*n* < 15)
Host response	Marked	Moderate	Slight	None
Nuclear pleomorphism	<25% anaplasia	25–50% anaplasia	50–75% anaplasia	75–100% anaplasia
Mitosis HPF * (40×)	0–1	2–3	4–5	>5

* HPF: high-power field.

**Table 2:** Tumor Cell Budding System used in our study for determining tumor grade based on tumor budding activity and cell next size score adapted from human cervical SCC (Jesinghaus et al. (2018)) and laryngeal/hypopharyngeal SCC (Boxberg et al. (2019)).

Tumor Budding Activity/10HPF	Score Value
No budding	1
<15 budding foci	2
≥15 budding foci	3
**Smallest cell nest size**	
>15 cells	1
5–15 cells	2
2–4 cells	3
Single cell invasion	4

## Data Availability

Data available on request due to restrictions, e.g., privacy or ethics.

## Supplementary Materials

The following supporting information can be downloaded at: https://www.mdpi.com/article/10.3390/vetsci10020088/s1.
