# Supplementary material for: Evaluating the Histologic Grade of Digital Squamous Cell Carcinomas in Dogs and Copy Number Variation of KIT Ligand—A Correlation Study
Source: Vet Sci. 2023 Jan 24;10(2):88. doi: 10.3390/vetsci10020088 (PMC9962207; doi:10.3390/vetsci10020088)
Supplement: Supplementary file 1 [file vetsci-10-00088-s001.zip › Supplemental table S1-1.pdf]

Sample Number	Breed	Age (years)	Gender	limb 0=fore- 1=hind-	side 0=right 1=left	toe	Groups 1.a= black; 1.b=Schnauzers; 1.c=Black&tan; 2=light; 0= other breeds	Nagamine, Invasive front grade	Jesinghaus, Cellular dissociation grade	CNV Average
1	Belgian Shepherd	16	fc	0	0	1	0	I	1	4,52
2	German Wirehaired Pointer	14	m	0	1	2	0	II	3	5,85
3	Hovawart	8	f	1	1	5	0	I	1	5,80
4	Labrador	8	f	0	1	3	0	I	3	4,66
5	Mixed	10	fc	0	0	5	0	II	2	5,03
6	Mixed	9	fc				0	I	1	5,19
7	Mixed	11	f	0	1	2	0	III	3	5,62
8	Mixed	13	mc	0	0	3	0	II	3	7,00
9	Mixed	6	m	0	0	5	0	II	2	4,91
10	Mixed	10	f	0	1	3	0	III	2	9,14
11	Mixed	9	fc	0	0	1	0	III	3	4,94
12	Briard	11	f	0	1	2	1.a	III	3	4,83
13	Briard	11	mc	1	0	1	1.a	II	1	5,91
14	Briard	7	mc	0	1	2	1.a	I	1	6,74
15	Briard	6	m	0	0	4	1.a	I	1	7,50
16	Flat Coated Retriever	8	m	0	1	3	1.a	I	1	5,31
17	Flat Coated Retriever	8	mc	0	0	5	1.a	II	3	5,98
18	Giant Standard Poodle	7	mc	0	1	3	1.a	I	1	4,68
19	Havanese	13	fc	1	1	5	1.a	II	1	4,76
20	Labrador	8	m	1	0	5	1.a	I	2	4,46
21	Labrador	11	mc	0	1	5	1.a	I	1	5,77
22	Labrador	9	m	1	1	5	1.a	I	3	5,75
23	Labrador	7	mc	0	1	5	1.a	II	3	6,41
24	Puli	12	mc	1	1	2	1.a	II	3	5,27
25	Russian Black Terrier	10	m	0	0	2	1.a	II	2	6,77
26	Russian Black Terrier	10	m	0	1	4	1.a	III	3	6,77
27	Standard Poodle	14	fc	0	1	5	1.a	II	2	5,45
28	Giant Schnauzer	12	f	0	1	4	1.b	II	1	6,68
29	Giant Schnauzer	10	f	1	1	4	1.b	II	3	5,79

30	Giant Schnauzer	9	mc	0	0	5	1.b	III	3	5,85
31	Giant Schnauzer	8	mc	0	0	5	1.b	I	3	5,35
32	Giant Schnauzer	11	mc	0	1	5	1.b	II	2	6,55
33	Giant Schnauzer	10	m	0	1	1	1.b	II	1	5,36
34	Giant Schnauzer	8	m	0		1	1.b	I	1	5,97
35	Giant Schnauzer	10	m	0	0	2	1.b	II	3	5,73
36	Giant Schnauzer	12	fc	1	0		1.b	II	2	6,65
37	Giant Schnauzer	12	fc	0	1	2	1.b	II	3	5,91
38	Giant Schnauzer	8	m				1.b	II	2	5,29
39	Giant Schnauzer	12	mc	0	1	2	1.b	II	2	5,81
40	Giant Schnauzer	10	mc	0	1	5	1.b	II	2	6,30
41	Giant Schnauzer	7	m	0	1	5	1.b	II	1	6,10
42	Giant Schnauzer	11	f	1	0	5	1.b	II	1	6,85
43	Giant Schnauzer	11	f	1	0	5	1.b	II	2	6,85
44	Giant Schnauzer	12	fc	0	1	3	1.b	II	1	4,75
45	Giant Schnauzer	6	m	0	0	2	1.b	II	2	5,30
46	Giant Schnauzer	11	f	1	1	5	1.b	I	1	5,59
47	Giant Schnauzer	9	mc	0	0	2	1.b	II	2	6,15
48	Standard Schnauzer	5	f	1	1	5	1.b	II	3	6,70
49	Standard Schnauzer	6	mc	0		4	1.b	I	1	5,21
50	Standard Schnauzer	8	fc	1	1	1	1.b	II	1	5,16
51	Standard Schnauzer	13	f	1	1	1	1.b	III	3	5,87
52	Standard Schnauzer	10	m	1	0	4	1.b	I	1	5,68
53	Standard Schnauzer	8	m	1	1	1	1.b	II	3	5,75
54	Standard Schnauzer	8	fc	1	0	5	1.b	I	1	4,44
55	Standard Schnauzer	12	mc	1	1	5	1.b	I	1	5,20
56	Standard Schnauzer	10	m	0	1	3	1.b	II	2	5,47
57	Standard Schnauzer	13	m	0	1	5	1.b	II	2	5,41
58	Standard Schnauzer	9	fc	0	1	5	1.b	I	1	5,40
59	Standard Schnauzer	10	fc	0	1	2	1.b	I	2	4,70
60	Standard Schnauzer	12	mc	0	0	1	1.b	I	1	5,20
61	Standard Schnauzer	11	mc	1	1	5	1.b	I	2	4,90
62	Bernese Mountain Dog	10	mc	0	0	1	1.c	I	1	4,13
63	Bernese Mountain Dog	6	f	0	0	3	1.c	I	1	5,87
64	Gordon Setter	14	mc	1	1	1	1.c	II	3	4,96

65	Gordon Setter	10	m	1	0	2	1.c	I	1	5,02
66	Gordon Setter	9	m	0	0	2	1.c	II	3	6,68
67	Rottweiler	11	m	0	0	4	1.c	I	1	5,45
68	Rottweiler	10	fc	1	1	3	1.c	III	3	6,35
69	Briard	8	m	1	0		2	II	1	5,30
70	Irish Red Setter	14	fc	0	1	4	2	I	1	5,73
71	Mixed	4	f	1	1	3	2	I	1	2,17
72	WHWT	12	f	0	1	1	2	I	1	4,98
