# Supplementary figures and images for: Evaluating the Histologic Grade of Digital Squamous Cell Carcinomas in Dogs and Copy Number Variation of KIT Ligand—A Correlation Study
Source: Vet Sci. 2023 Jan 24;10(2):88. doi: 10.3390/vetsci10020088 (PMC9962207; doi:10.3390/vetsci10020088)

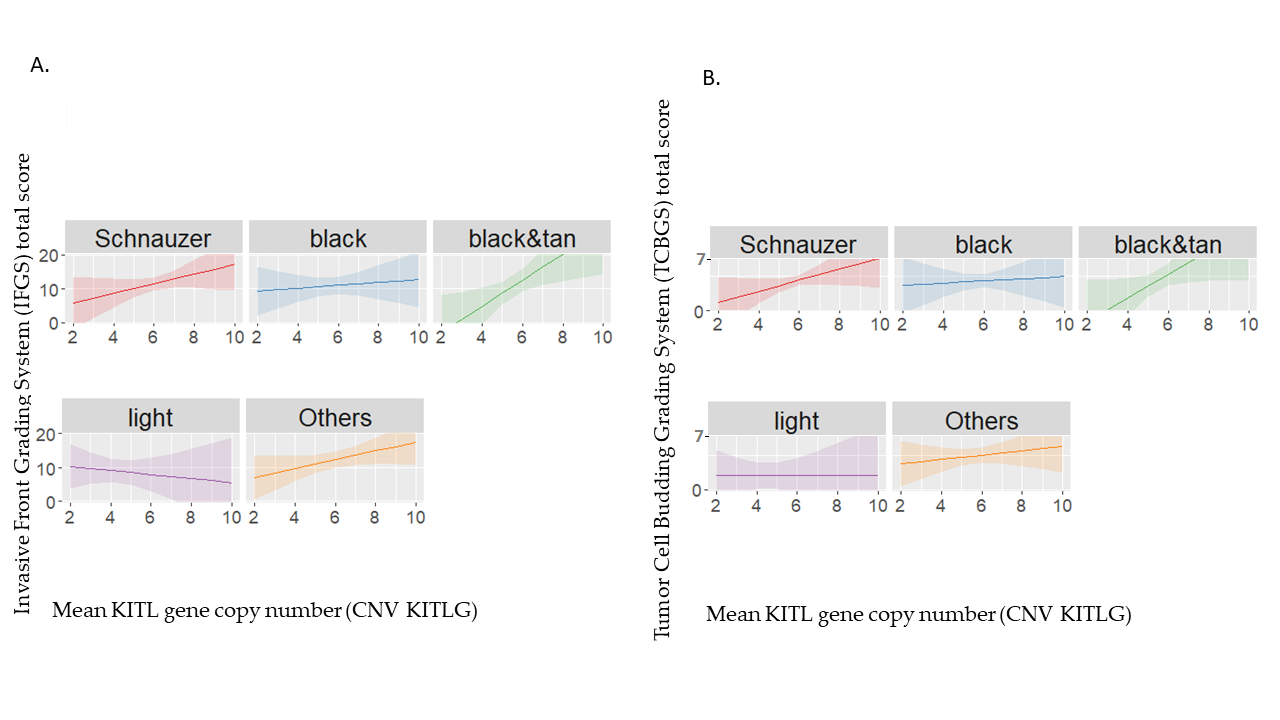

Supplement: Supplementary file 1 [file vetsci-10-00088-s001.zip › Supplemental Panel S1.jpg.png]
